# Late-Onset Angioimmunoblastic T-cell Lymphoma During Nivolumab Treatment for Melanoma

**DOI:** 10.7759/cureus.89042

**Published:** 2025-07-30

**Authors:** Akihiro Miyashita, Taichi Murao, Yuji Shimura, Haruya Okamoto, Hiroaki Nagata, Akio Onishi, Takahiro Fujino, Shinsuke Mizutani, Taku Tsukamoto, Junya Kuroda

**Affiliations:** 1 Department of Internal Medicine, John A. Burns School of Medicine, University of Hawai'i, Honolulu, USA; 2 Division of Hematology and Oncology, Kyoto Prefectural University of Medicine, Kyoto, JPN; 3 Department of Hematology, Panasonic Health Insurance Organization Matsushita Memorial Hospital, Osaka, JPN; 4 Blood Transfusion, Kyoto Prefectural University of Medicine, Kyoto, JPN

**Keywords:** angioimmunoblastic t-cell lymphoma, cholangitis, immune checkpoint inhibitors, immune-related adverse events, nivolumab

## Abstract

Immune checkpoint inhibitors (ICIs) are innovative immunotherapeutic agents used to treat various types of cancer by enhancing T-cell-mediated antitumor activity. These agents have distinct adverse events, known as immune-related adverse events, which can affect multiple organ systems and typically occur within a year after initiating ICI therapy. Another concern regarding ICI therapy is the potential development of T-cell lymphomas due to prolonged activation of T-cell activity. We report the case of a 56-year-old female who was treated with nivolumab for metastatic melanoma for over seven years and subsequently developed concurrent angioimmunoblastic T-cell lymphoma and ICI-related cholangitis. This case highlights the importance of careful monitoring for the emergence of late-onset T-cell lymphoma in patients undergoing long-term ICI therapy.

## Introduction

Immune checkpoint inhibitors (ICIs) are a groundbreaking class of immunotherapeutic agents that have significantly changed how various cancers are treated. Programmed cell death protein 1 (PD-1) is a receptor expressed on activated T cells, while its primary ligand, programmed death-ligand 1 (PD-L1), is expressed on tumor cells. When PD-L1 binds to PD-1, it delivers an inhibitory signal that suppresses T-cell activity. The most commonly used ICIs are PD-1/PD-L1 inhibitors, such as nivolumab and pembrolizumab, which block this costimulatory signal and thereby boost the T-cell-mediated antitumor response [1]. Due to their unique mechanism of enhancing immune function, ICIs are linked to a particular range of immune-related adverse events (irAEs) that can impact nearly any organ system [2-4]. Moreover, because these treatments block the inhibitory signals that regulate T-cell activity, they can lead to prolonged T-cell stimulation. This raises concerns that excessive and sustained T cell activation from extended ICI use may increase the risk of developing T-cell lymphoma [5], although supporting evidence remains limited.

In this report, we detail a rare case of late-onset angioimmunoblastic T-cell lymphoma (AITL) that occurred alongside ICI-related cholangitis, seven years following the initiation of treatment with nivolumab. While ICIs are primarily associated with early-onset irAEs, this report highlights the rare but potentially serious complications that can occur years after therapy begins.

This article was previously presented as a meeting abstract at the Japanese Society of Internal Medicine 2023 Kinki Regional Meeting, Osaka, Japan, on March 4, 2023.

## Case presentation

A 56-year-old Japanese female was initially diagnosed with advanced melanoma and lymph node metastasis involving the right scapular, right supraclavicular, and right cervical regions. She received nivolumab treatment, which led to complete metabolic remission within a year, with no significant side effects apart from the onset of vitiligo. The treatment continued for a total of seven years, during which she maintained her remission status. Seven years after the initiation of therapy, the patient developed pancytopenia and elevated liver function tests. Laboratory results indicated pancytopenia and elevated hepatobiliary enzymes (Table 1).

**Table 1:** Laboratory results at the initial visit ALT: alanine transaminase, AST: aspartate aminotransferase, CT: computed tomography, ICU: intensive care unit.

Laboratory test	Value	Reference range
White blood cells	3.5 x 10^9^/L	3.3-8.6 x 10^9^/L
Neutrophil	5%	39.8-70.5%
Lymphocyte	45%	23.1-49.9%
Eosinophil	26%	0.6-5.4%
Monocyte	23%	4.3-10.0%
Basophil	1%	0.3-1.4%
Red blood cell	3.69 x 10^6^/µL	3.86-4.92 x 10^6^/µL
Hemoglobin	9.9 g/dL	11.6-14.8 g/dL
Hematocrit	31.8%	35.1-44.4%
Platelet	102.0 x 10^9^/L	158-348 x 10^9^/L
Total protein	5.3 g/dL	6.6-8.1 g/dL
Albumin	2.2 g/dL	4.1-5.1 g/dL
Total bilirubin	1.90 mg/dL	0.4-1.5 mg/dL
AST	49 U/L	13-30 U/L
ALT	50 U/L	7-23 U/L
Alkaline phosphatase	572 U/L	38-113 U/L
Gamma-glutamyl transpeptidase	690 U/L	9-32 U/L
LDH	224 U/L	124-222 U/L
BUN	13.3 mg/dL	8.0-20.0 mg/dL
Creatinine	0.47 mg/dL	0.46-0.79 mg/dL

**Figure 1:**
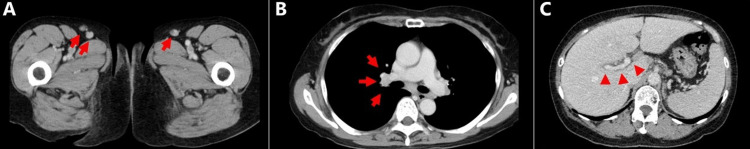
Radiological finding at the onset of angioimmunoblastic lymphoma. Computed tomography showed systemic lymphadenopathy (A and B, red arrows) and hepatosplenomegaly (C) with periportal edema (C, red arrowheads).

Bone marrow aspirate and biopsy showed moderate hypoplasia with a nucleated cell count of 46.0 × 10⁹/L and no signs of dysplasia or malignant lymphoma infiltration. Computed tomography demonstrated systemic lymphadenopathy, including mediastinal and inguinal areas, as well as hepatosplenomegaly associated with periportal edema (Figure 1).

**Figure 2:**
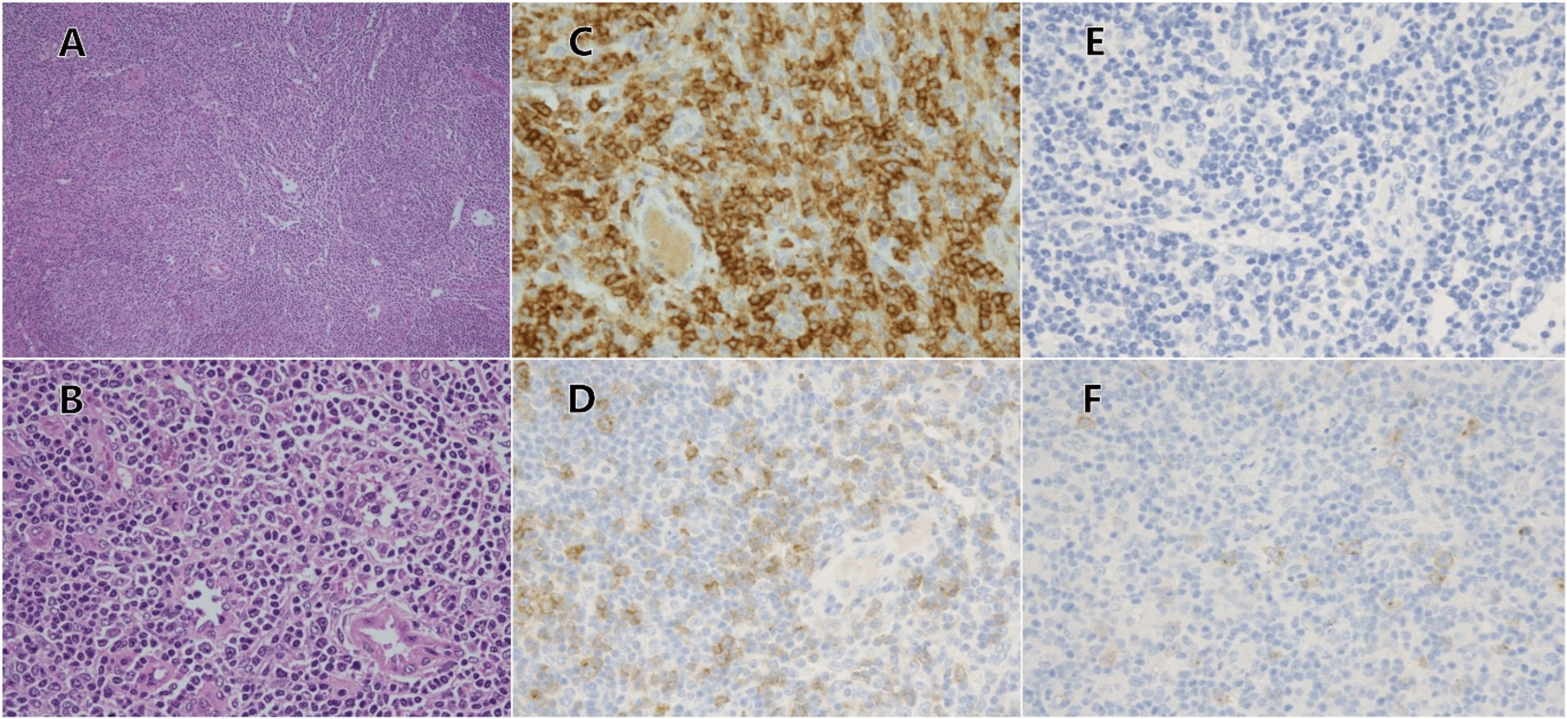
Histological and immunohistochemical views of the lymph node-biopsied specimen. The lymph node biopsy specimen showed proliferating medium- to large-sized lymphocytes (A, ×100 magnification; B, ×400; hematoxylin and eosin (HE) staining) that were positive for CD4 (C, ×400) and programmed cell death protein 1 (PD-1) (D, ×400), but negative for Epstein-Barr virus-encoded small RNA (E, ×400) and CD30 (F, ×400).

Lymph node biopsy showed atrophic germinal centers, with a notable increase in medium to large lymphocytes in the interfollicular regions, as well as enhanced venous structures. There was no evidence of eosinophil or plasma cell infiltration. The lymphocytes within the interfollicular areas were immunophenotypically identified as CD20⁻, CD3⁺, CD4⁺, CD5⁺, CD7⁺, CD8⁻, CD10⁻, and Bcl-6⁺, and demonstrated weak to moderate PD-1 positivity in approximately 80% of the cells, along with weak CD30 positivity. The MIB-1 labeling index was roughly 50% (Figure 2).

**Figure 3:**
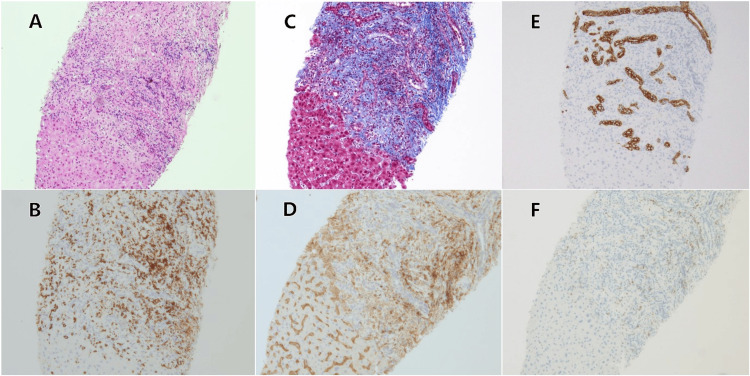
Histological and immunohistochemical views (x100 magnification) of liver-biopsied specimen. The specimen showed bile duct proliferation and portal fibrosis with lymphocyte infiltration, with no evidence of lymphoma cells (A, HE staining). Immunohistochemical staining revealed a predominance of CD8-positive cells (B), while CD4-positive cells were confined to the portal area (D). Masson's Trichrome stain demonstrated portal fibrosis (C), whereas Cytokeratin 7 expression was minimal (E). PD-1 expression was negative (F). These findings support a diagnosis of chronic cholangitis.

These observations were consistent with AITL. To assess liver dysfunction, a transjugular liver biopsy was performed. The portal regions exhibited fibrosis with lymphocyte and neutrophil infiltration, along with bile duct proliferation. Immunohistochemical staining highlighted a prevalence of CD8-positive cells, while CD4-positive cells were restricted to the portal regions. PD-1 staining yielded negative results (Figure 3).

These findings are consistent with chronic cholangitis caused by immune-related adverse effects from nivolumab. Subsequently, nivolumab was discontinued, and the patient received multi-agent chemotherapy, involving cyclophosphamide, doxorubicin, vincristine, and prednisolone (CHOP regimen). After six cycles, she achieved complete metabolic remission.

## Discussion

ICIs are innovative immunotherapy agents that greatly enhance the outlook for various cancer types [1]. Nivolumab, a PD-1 inhibitor, functions by blocking cancer cells from evading detection by T cells [6]. This treatment enhances T-cell activity to facilitate tumor destruction, which can lead to a diverse range of irAEs affecting multiple organ systems. The most commonly observed irAEs associated with PD-1/PD-L1 inhibitors include skin reactions, hormonal imbalances, and gastrointestinal issues [7]. Immune-mediated liver injury is a common irAE that may affect up to 25% of patients receiving ICIs. ICI-induced cholangitis represents a rarer subtype of immune-mediated liver injury, typically marked by a mixed or cholestatic pattern along with elevated alkaline phosphatase levels [8]. It is important to note that irAEs can occur at various intervals after the initiation of ICIs. For example, the median time to onset of skin rash is 6.1 weeks, GI symptoms 8.9 weeks, and endocrine side effects 12.0 weeks [7]. The median time to onset of ICI-related cholangitis has been reported to be 5.7 months, with a range of 3 to 28.8 months [8]. In our case, skin vitiligo developed soon after the initiation of nivolumab; however, seven years later, another irAE-ICI-related cholangitis occurred. To our knowledge, this is the first reported case of ICI-related cholangitis arising after long-term therapy, specifically seven years after initiation of treatment. The reason for the late-onset irAE in this case remains unclear; however, one retrospective analysis identified several predictors for early onset of irAEs, including female sex, combination therapy with chemotherapy, a pre-existing autoimmune condition, and a pre-existing infection [9]. In our case, apart from the patient’s sex, none of these risk factors were present, potentially explaining the delayed onset of the irAE.

AITL is a subtype of peripheral T-cell lymphomas (PTCLs), accounting for 15-20% of all PTCL cases and typically affecting middle-aged to older men. AITL originates from T follicular helper (TFH) cells, a specialized subset of CD4-positive T helper cells known for their high expression of PD-1 [10]. Immunophenotypically, AITL cells often express CD4 and PD-1, consistent with their TFH cell lineage. The pathogenesis of AITL is not fully understood; however, it may be related to several gene mutations, such as those in the ras homolog family member A (RHOA) or T-cell receptors (TCRs). The relationship between ICIs and the development of T-cell lymphomas remains unclear; however, several case reports have described T-cell lymphomas emerging after PD-1 inhibitor therapy (Table 2) [5,11-18].

**Table 2:** Previous reports of T-cell lymphoma development following treatment with immune checkpoint inhibitors ICI, immune checkpoint inhibitor; irAE, immune-related adverse event; Pembro, pembrolizumab; Nivo, nivolumab; ipi, ipilimumab; AITL, angioimmunoblastic T-cell lymphoma; SPTCL, subcutaneous panniculitis-like T-cell lymphoma; CTCL, cutaneous T-cell lymphoma; HSTCL, hepatosplenic T-cell lymphoma; PTCL-NOS, peripheral T-cell lymphoma, not otherwise specified; CHOP, cyclophosphamide, doxorubicin, vincristine, and prednisone; NA, not available.

Article	Age	Sex	Disease	ICI	Duration of ICI treatment (months)	irAE	Subsequent lymphoma	Treatment for lymphoma	Survival status	Survival period since lymphoma diagnosis (months)
Kawakami et al., 2022 [11]	68	M	Lung adenocarcinoma	Pembro	14	None	AITL	CHOP	Alive	1
Yu et al., 2024 [12]	53	M	Melanoma	Nivo	9	None	SPTCL	None	Dead	6
Hida et al., 2022 [13]	56	M	Melanoma	Nivo	18	None	CTCL	CHOP	Alive	5
Marks et al., 2021 [14]	53	M	Renal cell carcinoma	Nivo	12	None	CTCL	None	Dead	NA
Duke et al., 2020 [15]	78	M	Melanoma	Pembro	26	None	AITL	CHOP	Alive	20
Zheng et al., 2018 [16]	61	M	Melanoma	Pembro	21	Vitiligo	CTCL	Radiation and pralatrexate	Alive	NA
Ono et al., 2019 [17]	73	M	Classical Hodgkin lymphoma	Nivo	1	None	HSTCL	None	Dead	1
Koda et al., 2021 [18]	54	M	Lung adenocarcinoma	Nivo	16	Colitis	PTCL-NOS	CHOP	Dead	NA
Anand et al., 2020 [5]	70s	M	Undifferentiated carcinoma of epithelial origin	Pembro	3	None	PTCL-NOS	None	Dead	NA
Present case	56	F	Melanoma	Nivo	90	Vitiligo, cholangitis	AITL	CHOP	Alive	31

Reported latency periods from the initiation of ICI therapy to the development of lymphoma vary from 3 to 21 months, with some cases linked to concurrent irAEs. The reports differ regarding the type of primary cancer and T-cell lymphoma subtypes. Additionally, specific phase 2 trials noted rapid progression of adult T-cell leukemia/lymphoma and AITL following the administration of ICIs for the treatment of T-cell lymphoma [19]. Establishing a definitive link between ICIs and the development of T-cell lymphoma is challenging, but our case suggests that prolonged nivolumab use may have contributed to the emergence of AITL, particularly given the concurrent appearance of irAE-related cholangitis. The exact mechanism behind ICI-induced secondary T-cell lymphoma remains unclear; however, one plausible theory posits that ongoing immune hyperstimulation resulting from ICI use could favor the emergence of a clone that contributes to T-cell lymphoma development. Another hypothesis proposes that the PD-1 receptor within tumor cells may function as a tumor suppressor [20], indicating that blocking PD-1 could potentially promote tumor growth. Further research, including the investigation of gene mutations, is necessary to explore the relationship between these immune-related therapies and the development of T-cell lymphomas.

## Conclusions

In summary, we present a rare case of late-onset ICI-related cholangitis occurring alongside the development of AITL, seven years after initiation of nivolumab treatment. While most irAEs occur shortly after treatment initiation, delayed presentations can emerge even several years later. Furthermore, ICI therapy may be associated with an increased risk of T-cell lymphoma. With the increasing use of ICIs, it is imperative to recognize these delayed and uncommon complications. Although causality cannot be conclusively established, the temporal link between prolonged ICI treatment and the onset of T-cell lymphoma warrants further investigation. Additional research is needed to better understand this association.
